# Metabolomic and transcriptomic analyses provide insight into the variation of floral scent and molecular regulation in different cultivars and flower development of *Curcuma alismatifolia*

**DOI:** 10.1093/hr/uhae348

**Published:** 2024-12-12

**Authors:** Chao Song, Jingpu Tian, Dejin Xie, Shengnan Lin, Yingxue Yang, Xiaoni Zhang, Xuezhu Liao, Zhiqiang Wu

**Affiliations:** Kunpeng Institute of Modern Agriculture at Foshan, Shenzhen Branch, Guangdong Laboratory of Lingnan Modern Agriculture, Agricultural Genomics Institute at Shenzhen, Chinese Academy of Agricultural Sciences, Shenzhen 518124, China; Department of Architecture and Design, Hunan University of Science and Technology, Taoyuan Road, Yuhu District, Xiangtan 411201, China; Shenzhen Branch, Guangdong Laboratory of Lingnan Modern Agriculture, Key Laboratory of Synthetic Biology, Ministry of Agriculture and Rural Affairs, Agricultural Genomics Institute at Shenzhen, Chinese Academy of Agricultural Sciences, Shenzhen, China; Kunpeng Institute of Modern Agriculture at Foshan, Shenzhen Branch, Guangdong Laboratory of Lingnan Modern Agriculture, Agricultural Genomics Institute at Shenzhen, Chinese Academy of Agricultural Sciences, Shenzhen 518124, China; Kunpeng Institute of Modern Agriculture at Foshan, Shenzhen Branch, Guangdong Laboratory of Lingnan Modern Agriculture, Agricultural Genomics Institute at Shenzhen, Chinese Academy of Agricultural Sciences, Shenzhen 518124, China; Shenzhen Branch, Guangdong Laboratory of Lingnan Modern Agriculture, Key Laboratory of Synthetic Biology, Ministry of Agriculture and Rural Affairs, Agricultural Genomics Institute at Shenzhen, Chinese Academy of Agricultural Sciences, Shenzhen, China; Kunpeng Institute of Modern Agriculture at Foshan, Shenzhen Branch, Guangdong Laboratory of Lingnan Modern Agriculture, Agricultural Genomics Institute at Shenzhen, Chinese Academy of Agricultural Sciences, Shenzhen 518124, China; Shenzhen Branch, Guangdong Laboratory of Lingnan Modern Agriculture, Key Laboratory of Synthetic Biology, Ministry of Agriculture and Rural Affairs, Agricultural Genomics Institute at Shenzhen, Chinese Academy of Agricultural Sciences, Shenzhen, China; Shenzhen Branch, Guangdong Laboratory of Lingnan Modern Agriculture, Key Laboratory of Synthetic Biology, Ministry of Agriculture and Rural Affairs, Agricultural Genomics Institute at Shenzhen, Chinese Academy of Agricultural Sciences, Shenzhen, China; Kunpeng Institute of Modern Agriculture at Foshan, Shenzhen Branch, Guangdong Laboratory of Lingnan Modern Agriculture, Agricultural Genomics Institute at Shenzhen, Chinese Academy of Agricultural Sciences, Shenzhen 518124, China; Shenzhen Branch, Guangdong Laboratory of Lingnan Modern Agriculture, Key Laboratory of Synthetic Biology, Ministry of Agriculture and Rural Affairs, Agricultural Genomics Institute at Shenzhen, Chinese Academy of Agricultural Sciences, Shenzhen, China

## Abstract

*Curcuma alismatifolia* is an important ornamental plant of significant economic value, while the floral fragrance has been rarely investigated, leading to a lack of knowledge about the floral scent. By performing metabolomic and transcriptomic analyses, we investigated the variation of 906 volatile organic compounds (VOCs) in florets of eight *C. alismatifolia* cultivars and four different developmental stages of “Chiang Mai Pink” (CMP). The metabolite profiling revealed that the terpenoid group (213 out of 906) was the predominant VOC, accounting for 33.5% and 43.4% of total VOC contents in the florets of different cultivars and developmental stages, respectively. Sweet and woody were the predominant odors not only in different cultivars but also during developmental stages. The varied intensities of other odors contributed to forming odor diversities in *C. alismatifolia* floret. We uncovered seven terpenoid synthetase (*TPS*) genes and four *MYB* genes of significant association with the biosynthesis of terpenoids in eight cultivars and floret development, respectively. We performed an activity assay on four selected *TPS* genes and identified that Chr15HA1352 and Chr15HA2528 are responsible for the biosynthesis of *α*-farnesene. The significant association between the *MYB* gene (*Chr03HA28*) and seven terpenoids can be observed among different cultivars and during different developmental stages. These findings highlight the varying floral scents in different cultivars and floret development and suggest the potential roles of identified *TPS* and *MYB* genes in the biosynthesis of terpenoids in *C. alismatifolia*.

## Introduction

*Curcuma alismatifolia* is a tropical species belonging to Zingiberaceae family and widely distributed in the tropics of Asia to Africa and Australia [1]. It has a long history of being used as medicine due to the biological activity of turmeric and essential oil from rhizomes and leaves [2–4]. In addition to its pharmaceutical value, *C*. *alismatifolia* has gained increasing popularity as an ornamental cut or potted flower in China and Southeast Asia. It has a distinctive inflorescence comprising several large and colorful bracts and small flowers on a long peduncle. The considerable length of vase life facilitates the popularity of *C. alismatifolia* in the cut flower market [5]. Most recent studies have focused on the bract color of *C*. *alismatifolia*, the most obvious trait attracting the attention of breeders and researchers [6–8]. These studies identified a wide range of anthocyanins, main pigments, and related genes from *Curcuma* species and uncovered the formation and regulation of brilliant color in *C*. *alismatifolia*.

Besides bract color, floral scents also possess high ornamental and economic values to cut flower production in the floricultural industry [9–12]*.* Floral volatile organic compounds (VOCs) play crucial roles in attracting and guiding pollinators, as well as important traits for the quality of plants in the perfume, cosmetics, food, drink, and pharmaceutical industries [13]. It has been found that the Zingiberaceae family is rich in terpenoid compounds in different tissues, such as in rhizomes, leaves, and inflorescences [2, 3, 12]. In *C*. *alismatifolia*, our previous research revealed that terpenoids, such as sesquiterpenoids and monoterpenoids, are the dominant VOCs during flower development of “Chiang Mai Pink” [10]. Terpenoids are the most abundant group of VOCs, encompassing more than 40 000 individual compounds [14]. It possesses diverse functions due to its metabolite diversity. For instance, the emitted terpenoids can specifically attract targeted pollinators and avoid ineffective visitors depending on the context and abundance released by flowers [15–17]. Moreover, some terpenoids play important roles in forming flower fragrances, such as citronellol, geraniol, linaool, and nerol [18]. Those important terpenoids, individually or with other VOCs, can format different floral fragrances in different flower plants. For instance, most fragrant rose cultivars have great amounts of geraniol, nerol, and β-citronellol released by petals, and these terpenoids contribute to formatting a smell of essential oil of rose [19, 20]. Moreover, terpenoids and their derivatives vary in different species and varieties [21]. VOCs have been comprehensively documented in different ornamental flowers, while the volatile profile in *C*. *alismatifolia* flower is rarely reported.

Terpenoids encompass huge amounts of volatiles, and there are enormous variations in the types and amounts of terpenoids produced by different species. However, the basic terpenoid biosynthetic pathways are common in land plants, which are known to be biosynthesized from the mevalonic acid (MVA) pathway in cytosol and methylerythritol phosphate (MEP) pathway in plastids [22]. In MVA and MEP pathways, terpene synthases (TPSs) are pivotal enzymes that initiate terpenoid biosynthesis by using different precursors as substrates and converting them into different terpenoids [23]. Based on sequence homology, seed plant TPSs have been clustered into seven subfamilies, of which TPS-a, TPS-b, and TPS-g from angiosperm plants are responsible for floral volatile terpenoids, while TPSs responsible for terpene synthesis in gymnosperm were grouped into TPS-d family [24–26]. These identified *TPS* genes have shown their temporal and spatial expression in plants, resulting in volatile diversity between different tissues, developmental stages, and cultivars [27–31]. Those studies on the specific function or regulation role of TPSs in the biosynthesis of terpenoids can facilitate the understanding of how floral scent formats in ornamental plants. In addition to the *TPS* gene, transcription factors (TFs) are another group of genes that regulate the biosynthesis of terpenoids. Those TFs, such as basic helix–loop–helix (bHLH), WRKY, ethylene-responsive factor (ERF), and MYB, can regulate the emission of terpenoids by binding to the promoter regions of biosynthetic genes [18, 23, 32]. Among those TFs, MYB TFs are the major TFs contributing to the regulation of terpenoid biosynthesis [18, 23]. MYB proteins have a conserved N-terminal region (MYB DNA-binding domain) and a diverse C-terminal modulator region responsible for the regulatory activity. MYB TFs can be classified into four subfamilies based on the number of adjacent repeats in the conserved DNA-binding region: 1R-MYB (MYB-related), R2R3-MYB, 3R(R1R2R3)-MYB, and 4R-MYB proteins [33]. Within the MYB subfamilies, R2R3-MYB proteins are the most abundant group in plants and have been documented to show diverse functions in regulating the biosynthesis of volatile aroma production [34–36]. The plants with silenced MYB TFs presented decreased expression of *TPS* genes, accompanied by a significant change in the contents of terpenoids [37, 38]. Those findings greatly assist in exploring the regulation of *TPS* genes and TFs in terpenoid biosynthesis and floral scent.

The floral scent and regulation mechanism of specific fragrant metabolite has been widely and well investigated to assist in breeding new varieties of economic values in ornamental flower plants. However, the knowledge of flora scent in *C*. *alismatifolia* is lacking, and more effort needs to be made to uncover the fragrance characteristics and possible mechanisms to promote its economic value in the cut flower market. In this study, we performed metabolite profiling on the floral scent to shed light on the variation of floral volatiles among different cultivars and developmental stages of *C*. *alismatifolia* flower. In addition, transcriptomic analysis was conducted and integrated with metabolomic data to reveal the potential regulators in the flora scent of *C*. *alismatifolia*. The findings of this research will lay the knowledge foundation of the floral scent of *C*. *alismatifolia* and provide genetic assistance in the breeding of new cultivars with improved floral fragrance and economic value.

## Results

### Metabolite profiling on VOCs reveals the variation in flower scent of eight *curcuma alismatifolia* cultivars

**Figure 1:**
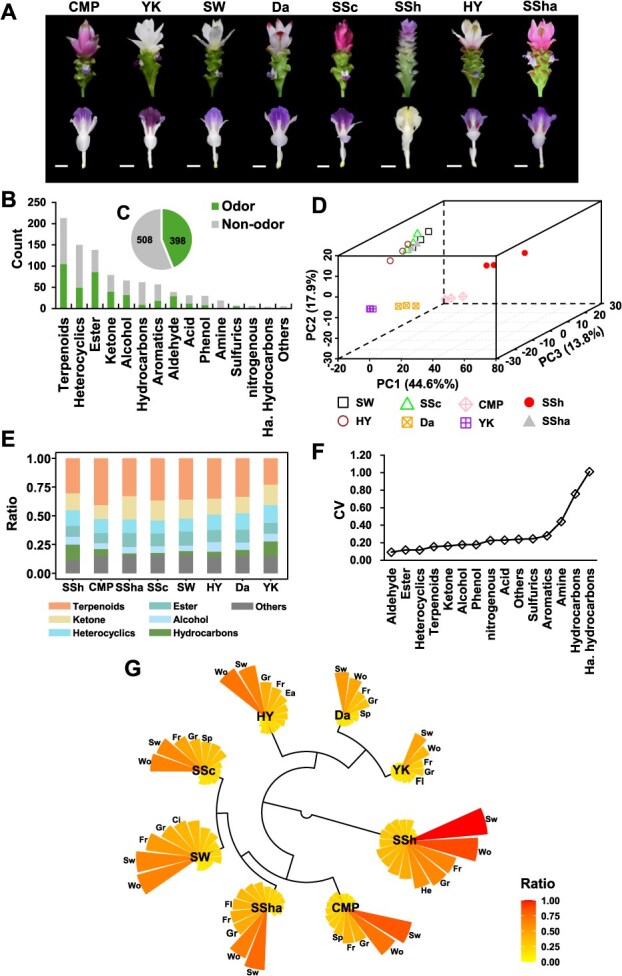
The metabolite profiling in florets of eight *C. alismatifolia* cultivars. **A**, the photograph of inflorescence (first row) and floret (second row) of eight *C. alismatifolia* cultivars. Each white bar under the floret indicates 1 cm. **B**, the metabolite classes derived from 906 VOCs based on their structures. **C**, the number of VOCs with odor and without odor. **D**, principal component analysis on 906 VOCs in eight cultivars. **E**, proportions of metabolite classes derived from 906 VOCs; the top six classes were shown, and the rest were summarized as the “other” group. **F**, The coefficient of variation of metabolite classes across eight cultivars. **G**, the odor types and relative intensity in the florets of each cultivar, the top five odor types were labeled, and more details were shown in supplementary table 1.

Based on metabolite profiling, a total of 906 VOCs were identified across eight cultivars, and these VOCs were assigned to 14 metabolite classes and one “other” group (Fig. 1B and C). The metabolite profiling on VOCs revealed varietal variations that four cultivars (“SW,” “HY,” “SSC,” and “SSha”) closely distributed, while the other four cultivars showed great differences, as illustrated by the PCA plot **(**Fig. 1D). Among VOCs, terpenoids are the largest class (213 out of 906) in the examined cultivars, averagely accounting for 33.5% of total intensity. “CMP” was found to have the highest terpenoid ratio (41.0%), whereas “YK” was the cultivar with the lowest terpenoid ratio (22.9%). The top four VOC classes (terpenoids, heterocyclics, esters, and ketones) represent, on average, 72.9% of the total VOCs in eight cultivars (Fig. 1E). The results on metabolite classes indicate the consistency of major VOCs across eight cultivars. We calculated the coefficient of variation (CV) on the ratio of VOC response of each class to total VOCs across eight cultivars, revealing the large variabilities for halogenated hydrocarbons with CV value 1.01 (Ha. Hydrocarbons) across cultivars. The terpenoid, heterocyclic, and ester groups had the lowest CV values (Fig. 1F), and these results suggest the stability of those VOC groups across different cultivars.

By calculating the abundance of each odor type of VOCs, we found that four odor types, including woody (Wo), sweet (Sw), green (Gr), and fresh (Fr), were the dominant odors in all eight cultivars, while the intensity of each odor type varied greatly among them (Fig. 1G and Table S1). “SSH” showed the highest intensities of four dominant odors than other cultivars, with extraordinary odors of sweet and woody. “SSC,” “SW,” “SSha,” and “CMP” presented similar patterns in the intensity of four dominant odors, indicating the similar floral scent of these cultivars. The rest cultivars (“Da,” “YK,” and “HY”) displayed a relatively lower intensity of dominant odors among the tested cultivars. For instance, the intensity of sweet odor in “Da” and “YK” was 53% and 48% of that in “SSh”, respectively (Fig. 1G, Table S1).

### WGCNA identifies the hub genes associated with terpenoid biosynthesis

**Figure 2:**
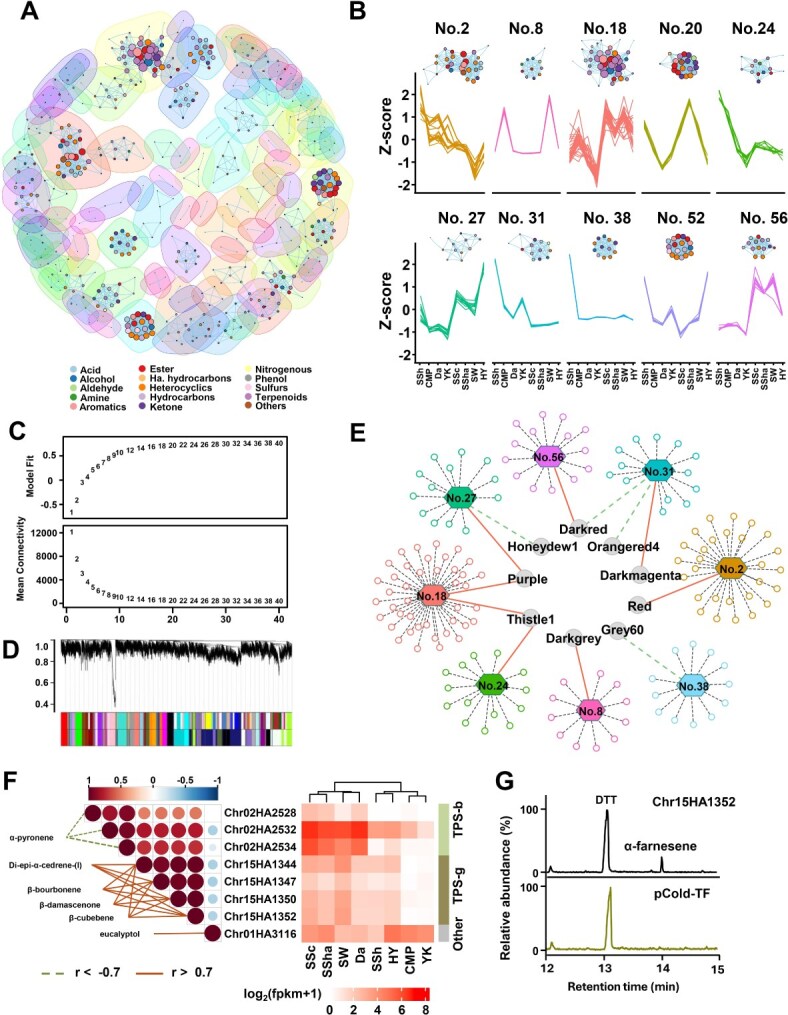
Integration analysis revealed the *TPS* genes of potential associations with terpenoid biosynthesis. **A**, Network of significant correlations (*q*-value <1.0 × 1e^−10^) between VOCs. The communities of VOCs were shown with an outlined background based on the connectivity between VOCs. **B**, relative levels of VOCs in each community across eight cultivars. The top ten communities with the highest number of VOCs were shown. **C**, the selection of soft power based on the scale free topology model fit (left) and mean connectivity (right). **D**, hierarchical clustering tree based on co-expression network. Each leaf in the tree represents one gene. **E**, the significant correlation (*q* < 0.01) between VOC communities (hexagon) and merged modules (grey circle) from WGCNA. The solid and dashed edges between communities and modules indicate positive and negative correlations, respectively. The hexagons and circles represent the VOC communities and the corresponding volatile metabolites of each community, respectively. **F**, the expression of selected genes (heatmap, right) and their correlations with each other (triangle heatmap, middle) and terpenoids connected by dashed lines. The solid and dashed lines indicate positive and negative correlations (|r| > 7), respectively. **G**, Relative contents of enzymatic products of selected *TPS* genes using (*E*, *E*)-FPP as substrate.

To uncover the possible genetic control of VOCs in different cultivars, we used all the genes and VOCs of strong association to perform WGCNA. The identified VOCs were correlated with each other across eight cultivars, and the significant correlations (*q*-value <1.0 × 1e^−10^) derived from 548 VOCs were obtained and visualized as a network (Fig. 2A). The top 10 communities with the highest number of members, including 184 VOCs in total, were obtained presenting different patterns in the levels of VOCs across eight cultivars (Fig. 2B), indicating high coordination of the VOCs within each community. Based on the highly associated changes of VOCs in each community, the average change of VOCs in each community was calculated to represent the response of the VOC community across eight cultivars (Fig. 2B).

23 374 expressed genes were fed into WGCNA (Fig. 2C and D), resulting in 37 merged modules (similarity >80%) from 60 modules (Fig. 2D). Then, we correlated the 37 merged modules with the average VOC community response, resulting in significant correlations (|r| > 0.78 and *q* < 0.01) between nine gene modules and eight VOC communities (Fig. 2E), revealing their potential role in the VOC biosynthesis. To identify the hub genes associated with the VOC community, we estimated correlations between module membership (MM) and gene significance (GS) values. With the criteria of MM > 0.8 and GS > 0.7, 1193 hub genes were identified from different modules. By performing the Kyoto Encyclopedia of Genes and Genomes (KEGG) enrichment analysis with those hub genes, the term “monoterpenoid biosynthesis” was enriched with eight hub genes (Fig. 2F), of which three genes (*Chr02HA2528*, *Chr02HA2532*, and *Chr02HA2534*) belong to *TPS-b* subfamily, four genes (*Chr15HA1344*, *Chr15HA1347*, *Chr15HA1350*, and *Chr15HA1352*) belong to *TPS-g* subfamily, and one gene (*Chr01HA3116*) was related to the biosynthesis of (+)-Neomenthol (Fig. 2F). The genes of each *TPS* subfamily were highly correlated with each other, as well as with some terpenoids (Fig. 2C, left). Based on the expression pattern and sequence analysis, we further selected four genes (*Chr02HA2528*, *Chr02HA2532*, *Chr02HA2534*, and *Chr02HA1352*) from *TPS-b/g* subfamily for functional characterization (Fig. 2G and Fig. S2). When (*E*, *E*)-FPP was used as a substrate for in vitro assays of the four TPS enzymes, Chr15HA1352 was shown to be a sesquiterpene synthase, which catalyzed the formation of a single product *α*-farnesene (Fig. 2G). The Chr02HA2528 recombinant protein produced a similar product to Chr15HA1352, presenting *α*-farnesene and *γ*-bisabolene as its main products. In comparison, the remaining two recombinant proteins, Chr15HA2534 and Chr15HA2532, with (*E*, *E*)-FPP substrate generated β-farnesene and farnesol as products, respectively. These results indicated that multiple *TPS* genes (*Chr15HA1352* and *Chr02HA2528*) are involved in the biosynthesis of sesquiterpene *α*-farnesene, the main components of *C. alismatifolia* floral scent.

### Metabolite profiling on volatile compounds at different developmental stages of CMP floret

**Figure 3:**
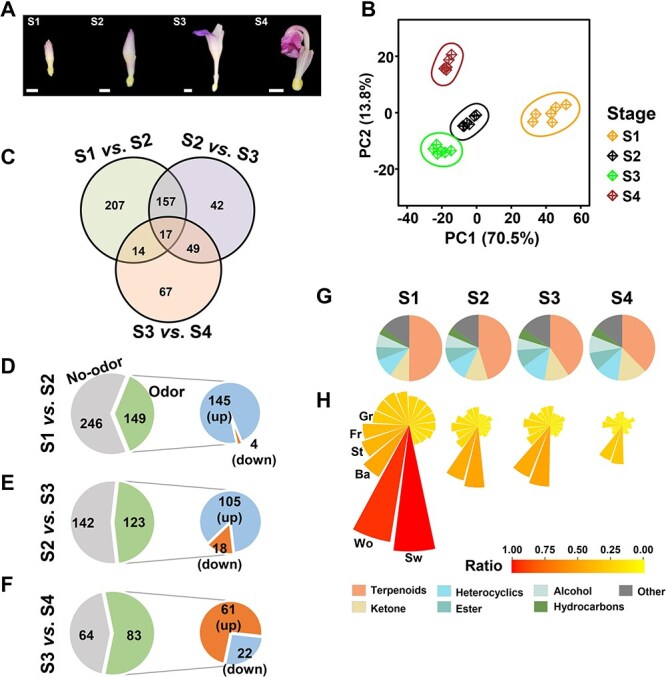
The metabolite profiling in florets of different developmental stages of CMP flower. **A**, the photograph of four typical developmental stages of “CMP” floret. Each white bar under the floret indicates 0.5 cm. **B**, principal component analysis on VOCs in florets. **C**, the number of DCVOCs (*q* < 0.05 and |log_2_ FC| > 1) between different stages. **D–F**, the bigger pie plots in each subplot showed the number of DCVOCs annotated with odor and non-odor. The smaller pie plots display the number of differentially changed (*q* < 0.05) odor VOCs with higher (blue) or lower (orange) contents than the next stage. **G**, the top six VOC classes are calculated as the abundance of VOC in each class relative to the total abundance of VOCs. **H**, the primary VOC odors at different stages of “CMP” florets. The top six odors were labeled: Sw, sweet; Wo, woody; Ba, balsam; St, storax; Fr, fruity; Gr, green, and the order of these odors is consistent across four stages.

To gain knowledge on VOCs during floret development, the florets of four typical developmental stages were collected for volatile metabolite profiling (Fig. 3A), and 896 VOCs were identified across four developmental stages after filtering out low levels of VOCs. The PCA, representing 84.3% of the total variation, clearly distinguished floret samples by developmental stages (Fig. 3B). During the floret development, the highest total abundance of VOCs was observed at the S1 stage and sharply reduced at the S2 stage (Fig. S1B), yielding 395 differentially changed VOCs (DCVOCs) (Fig. 3D–F). After the floret blooming, the abundance of VOCs decreased to the lowest level compared to the other developmental stages (Fig. S1B). Among the DCVOCs, most of the VOCs with odor were reduced from S1 to S2 (145 out of 149, Fig. 3D) and S2 to S3 stages (105 out of 123, Fig. 3E), suggesting the reduction of floral scent as floret developed. On the contrary, more than 73% of VOCs with odor significantly (*q* < 0.05) accumulated to higher levels from S3 to S4 stages (Fig. 3F).

When considering the VOC classes during floret development, terpenoids, ketones, heterocyclic compounds, and esters remain the dominant VOC classes, accounting for, on average, 43.4%, 11.9%, 10.9%, and 8.0%, respectively (Fig. 3G). The proportion of terpenoids greatly decreased by 12.5% from S1 (50.1%) to S4 (37.6%). In contrast, the proportion of ketones gradually increased from 9.5% to 14.4%, along with floret development. Regarding the floral odor, sweet (Sw) and woody (Wo) odors are primary floral scents during floret development. Flowers at the initial stage (S1) presented intensely sweet and woody odors, much higher than others (Fig. 3H). The intensity of floral odor distinctly dropped as the floret developed, and the abundance of primary odors (sweet and woody odors) at S4 was around 30.5% and 31.4% of that at S1, respectively (Table S2). Moreover, the pattern of major floral scents varied during floret development. For instance, the proportions of fruity (Fr) and green (Gr) odors were lower than that of balsam (Ba) and storax (St) at S1, while the pattern was reversed at S4 (Fig. 3H).

### Identification of VOCs related regulators during different floret stages

**Figure 4:**
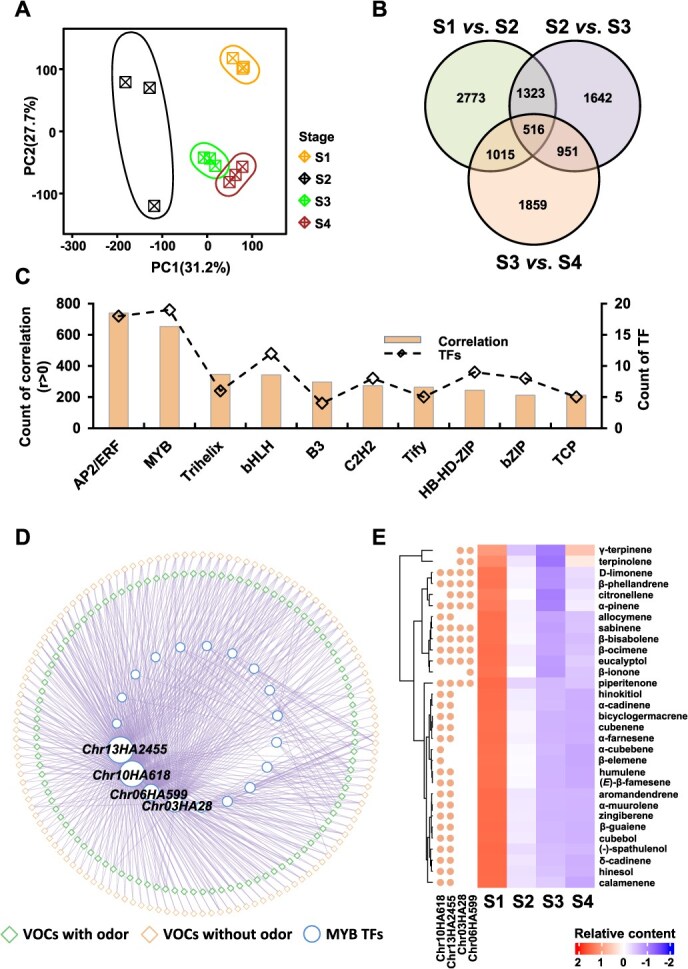
Integration analysis revealed the MYB transcription factors of potential associations with terpenoids biosynthesis in floret development. **A**, principal component analysis on gene expression in florets during different developmental stages. **B**, the number of DEGs (*q* < 0.05 and |log_2_ FC| > 1) between different stages. **C**, the top 10 transcription factor families descending ranked by the number of correlations with VOCs. **D**, the network of significant correlations (*q* < 0.05) between DCVOCs and MYB transcription factors. **E**, the relative contents of terpenoids during floret development (heatmap, right). The circle annotations at the left side of the heatmap indicate significant (orange) correlations (*q* < 0.05) between corresponding MYB TFs and terpenoids.

To search for possible regulatory genes associated with flower scent during floret development of “CMP,” we performed transcriptomic analysis on gene expression in florets from S1 to S4 stages, presenting distinct expression patterns between different stages (Fig. 4A). Along with the floret development, 5627, 4432, and 4341 differentially expressed genes (DEGs) were obtained from the comparisons between neighboring stages, namely S1 *vs.* S2, S2 *vs.* S3, and S3 *vs.* S4, respectively (Fig. 4B). To further address the association between DEGs and VOCs, the overlapping DEGs between comparisons were isolated, resulting in 3805 DEGs. Then, those 3805 DEGs were correlated with 237 DCVOCs, isolated from the overlaps of diagrams (Fig. 3B). The correlation analysis between selected DEGs and VOCs yielded 90 432 significant correlations (*q* < 0.05), of which 10 691 were related to 316 *TF* genes. Among those *TF* genes, 191 genes of 50 TF families were identified with high and positive (r > 0.75 and *q* < 0.05) associations with DCVOCs during floret development (Table S3). AP2/ERF (APETALA2/ethylene-responsive element binding factors) and MYB (or MYB-related) families were observed with the highest counts of correlations, showing 739 and 653 significant correlations from 18 AP2/ERFs and 19 MYBs, respectively (Fig. 4C). As MYB TFs are of high potential in regulating secondary metabolism and floral scent [23, 39], we furtherly focused on the correlations between *MYB* genes and corresponding VOCs. The correlation-based network highlighted the intense connections between four *TF* genes (*Chr13HA2455*, *Chr10HA618*, *Chr06HA599*, and *Chr03HA28*) and 234 VOCs, of which 92 VOCs were annotated with various odors (Fig. 4D). When classified those 92 VOCs into different metabolite classes, terpenoids were found to be the main VOC class, consisting of 31 terpenoids from mono- and sesquiterpenes (Fig. 4E and Fig. S3). These terpenoids showed varied associations with the identified four MYB TFs. For instance, two *MYB* genes (*Chr13HA2455* and *Chr10HA618*) were generally correlated to most terpenoids (29 out of 31), and eight terpenoids were found to be highly associated with all four *MYBs*, such as α-pinene, limonene, *β*-ocimene, and eucalyptol. One terpenoid, *β*-ionone, was significantly (*q* < 0.05) associated with one *MYB* gene, *Chr06HA599* (Fig. 4E).

### The cross verification of isolated genes in different cultivars and floret development

**Figure 5:**
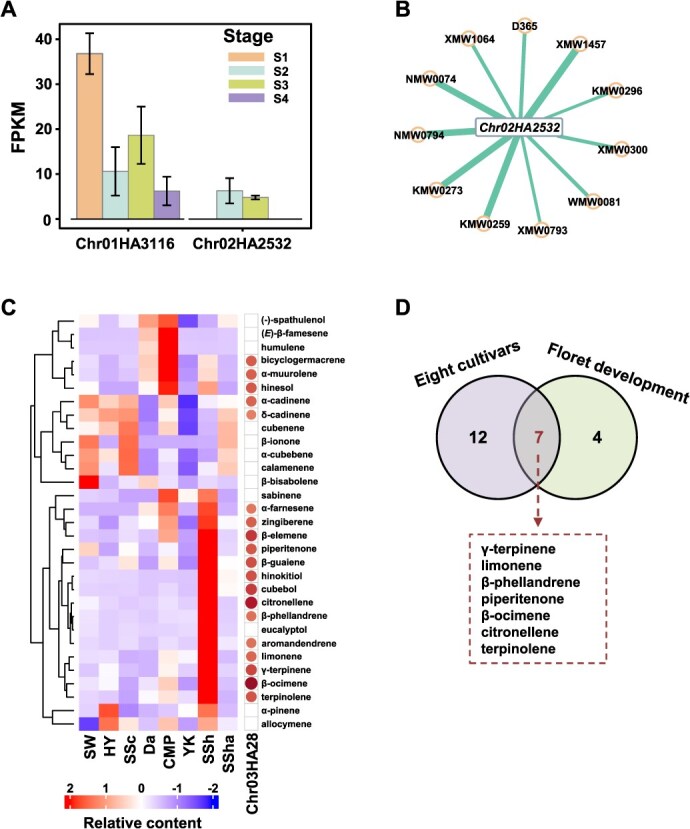
The associations of selected genes and VOCs in florets of eight cultivars and different developmental stages. **A**, the expression of two *TPS* genes, identified in eight cultivars, during floret development. **B**, the significant correlations (*q* < 0.05) between *Chr02HA2532* and 11 terpenoids in developmental florets. The metabolite IDs were shown, and more details were listed in supplementary table 4. **C**, the relative contents of selected terpenoids and their correlation with *MYB* gene, *Chr03HA28*. Small circles indicate significant (*q* < 0.05) and positive correlations (r > 0) between *Chr03HA28* and selected terpenoids. Insignificant correlations were filtered out and filled in blank. The larger and darker in circle size and color, the higher the correlation coefficient. **D**, the number of selected terpenoids that were significantly (*q* < 0.05) correlated with *Chr03HA28* in both eight cultivars and floret development.

As we identified the genes with potential association with VOCs of terpenoids in different cultivars and floret development (Figs 2F and 4E), then we checked if these genes have similar expression patterns in both conditions. First, we extracted the expression of eight genes identified from eight cultivars, while only two genes (*Chr01HA3116* and *Chr02HA2532*) were observed with relatively high expression in the floret development of “CMP” (Fig. 5A). As *Chr01HA3116* and *Chr02HA2532* were possibly related to terpenoid biosynthesis, the terpenoids of VOCs with significant correlation to the two genes were isolated, showing that 11 terpenoids were negatively associated with the expression of *Chr02HA2532* (r < −0.7 and *q* < 0.05) (Fig. 5B and Table S4). However, these correlations between *Chr02HA2532* and terpenoids observed from floret development did not overlap with those of different cultivars.

During floret development, we obtained four MYB TFs associated with 31 terpenoids (Fig. 4E). We examined the association between selected MYB TFs and terpenoids in eight *C. alismatifolia* cultivars (Fig. 5C). A pattern with higher contents of terpenoids were highlighted in “SSh” (18 out of 31) and “CMP” (6 out of 31). For instance, the relative contents of α-pinene, limonene, β-ocimene, and eucalyptol were much higher in “SSh” than in other cultivars. “CMP” has greater amounts of (−)-spathulenol, (*E*)-*β*-famesene, humulene, bicyclogermacrene, α-muurolene, and hinesol in florets than other cultivars. The significant correlations were observed only between one MYB gene (*Chr03HA28*) and 19 terpenoids (Fig. 5C), of which seven (such as limonene and *β*-ocimene) showed similar associations with the expression of *Chr03HA28* during floret development (Fig. 5D), suggesting that *Chr03HA28* may play an important role in the synthesis of the seven terpenoids.

## Discussion

### The diversity of floral scent in different *C*. *Alismatifolia* cultivars and floret development stages

Floral scents are a mixture of structurally diverse VOCs, usually classified as terpenoids, phenylpropanoids/benzenoids, fatty acid derivatives, and amino acid derivatives based on their original biosynthesis [22, 40]. Although *C*. *alismatifolia* has a long cultivation history in Asia as an ornamental plant with a sensory floral scent from florets [10], further exploration of its floral scent remains rarely mentioned. In the present study, non-targeted metabolomics was performed to investigate the variety and quantity of VOCs, illustrating that the variation was observed not only between cultivars but also in floret developmental stages.

Terpenoids are the largest class of plant VOCs as it comprises more than 40 000 structures from monoterpenoids, sesquiterpenoids, apocarotenoids, and others [18]. Totally, we identified 906 VOCs and classified them into 14 metabolic classes according to their structures (Fig. 1B). Our research showed that terpenoids were also the dominant VOCs in the florets of different cultivars and developmental stages (Figs 1E and F and 3G and Fig. S1A and B), which is consistent with the previous findings that terpenoids are the major VOCs in rhizomes, leaves, and inflorescences in Zingiberaceae family [2, 3, 12]. Although the variations exist in VOC contents across cultivars and between developmental stages, the predominance of terpenoids (22.9%–41.0%) remained in all examined samples, unlike the changed predominant VOC class in other ornamental plants with floral fragrance, such as rose [20], gardenia [41], tulip [42], and wild *Clematis* [43]. It was reported that the varied emission of major VOCs may reflect the adaptation strategies to the local pollinators, attracted by different floral attractants [23, 44, 45]. Our findings may suggest that the *C*. *alismatifolia* attracts some specific pollinators, such as bees [46].

The eight *C*. *alismatifolia* cultivars exhibited two patterns with respect to relative contents of VOCs, presenting a general similarity between four *C*. *alismatifolia* cultivars (“SW,” “HY,” “SSc,” and “SSha”) and a great variation between the rest of cultivars (“CMP,” “SSh,” “Da,” and “YK”), illustrated by sample distribution and abundance variations (Fig. 1D and Fig. S1A). The tremendous diversity of VOCs was commonly found in different species or cultivars of plants [47]. For example, roses have been cultivated and hybridized extensively, and different species or cultivars differ greatly in scent composition with different sensory aromas [48]. The total abundance of VOCs greatly varied across eight cultivars, which provides a guide for floral improvement and molecular breeding of *C*. *alismatifolia*.

Floral volatile compounds were synthesized and stored in volatile-producing cells and emitted during flower developmental changes [49]. Some compounds are bound to sugars as glycosides or glucosinolates, usually greater than free volatiles [50]. It has been reported that glycosidically bound volatiles are potentially a major source of aroma in flowers [51, 52]. In the flowers of *Osmanthus fragrans*,** the monoterpene volatiles were enhanced by β-D-glucosidase hydrolysis, indicating that the bound volatile aglycones are potential sources of floral compounds. The results showed that the initial flower development stage (Fig. 3A and Fig. S1) released the most abundant free VOCs than at later developmental stages in “CMP” (Fig. S1B). This characteristic is unlike other ornamental flowers, such as rose [53–55], orchid [56, 57], jasmines [58], and *Phalaenopsis-type Dendrobium hybrids* [59] showing the increased intensity of floral scent functioning as an attractant to potential pollinators during floret development. The condensed free VOCs inside the bud may not still transform to bound volatiles or, in turn, restrict the release of VOCs from volatile-producing cells into the atmosphere, leading to enrichment in floret tissues (Fig. S1B). During floret opening, the total abundance of free volatiles depleted from bud to blooming stages (S1–S3 stages, Fig. S1B), which indicates the characteristic of “CMP” floret that volatiles-producing decreases along with flower opening. However, more work remains to be done to investigate the quantity of bound volatiles during floret development.

Whatever these volatile compounds are produced in trace or high abundant amounts, which cannot be detected below the thresholds of human olfaction. Thus, we roughly summarized the dominant odors in eight different cultivars and developmental stages of “CMP” floret. The dominant odors were similar between cultivars and between different developmental stages, suggesting the consistent type of odors across eight cultivars and floret development. Meanwhile, the varied intensities of major odors and some non-dominant odors but highly sensed together can enrich the diversity of scents from floret and could help in distinguishing different cultivars (Figs 1G and 3H and Fig. S1). However, due to the diversity and different sensory thresholds, the major compounds of floral fragrance are still not clear, and more effort is needed to investigate the formation of floral scent in *C*. *alismatifolia*.

### The molecular mechanism of aroma terpenoid compound biosynthesis in different *C*. *Alismatifolia* cultivars

The molecular mechanism of floral scent is complicated as the floral volatile compounds biosynthesized from different pathways. Terpenoid compounds are major floral aroma constituents and play important roles in floral traits between different *C*. *alismatifolia* cultivars. However, the genes involved in the biosynthesis of floral VOCs in *C*. *alismatifolia* have been rarely reported. In this study, we performed pairwise correlations between VOCs and constructed a correlation-based network with highly associated VOCs, of which specific VOCs forming particular communities were obtained across eight cultivars (Fig. 2A and B**)**. Moreover, the community of coordinated VOCs was treated as a single volatile trait and fed into WGCNA with gene expression, facilitating the linkage between VOC communities and potential regulators. Consequently, integrating VOC profiles and gene expression by performing WGCNA revealed the interesting association between communities and gene modules (Fig. 2E). Notably, KEGG enrichment analysis on hub genes, resulting from WGCNA, highlighted eight genes enriched in the biosynthesis of monoterpenoids, of which three and four genes belong to two *TPS* subfamilies *TPS-b* and *TPS-g* (Fig. 2F), respectively. TPSs are the key enzymes for the biosynthesis of terpenoids, mediating temporal and spatial emission of multifarious VOCs in plants [27–31]. *TPS* genes display a diversity of family numbers and functions among different subfamilies. In land plants, they form a midsized gene family with striking functional complexity [25]. For instance, the *TPS* genes are usually multiple product and substrate enzymes, combined with the different subcellular localization of enzymes and further modification of enzymes, contributing to the greater diversity of terpene metabolism [24, 60]. In this study, the identified TPS-g subfamilies are related to the synthesis of some mainly sesquiterpenoids, such as *β*-cubebene, *β*-bourbonene, and *β*-damascenone (Fig. 2C). However, those two identified different *TPS* genes (*Chr15HA1352* and *Chr02HA2528*) are responsible for the same compound *α*-farnesene (Fig. 2G and Fig. S2). There were probably multiple enzymes and multiple products, therefore, the associated terpenoids and TPSs did not all necessarily correspond to each other. This result suggests the potential roles of *TPS* genes in the synthesis of terpenoids. At the same time, more efforts need to be made to investigate the biosynthesis of VOCs in *C*. *alismatifolia* cultivars.

### The MYB transcription factor genes are involved in regulating terpenoid biosynthesis during *C*. *Alismatifolia* flower development

TF plays a crucial role in regulating aroma-related volatiles in plants [18, 23, 61]. Terpenoids have multiple important biological functions and precise regulation mechanisms [62]. Therefore, it is acceptable that there are multiple TFs associated with terpene synthesis. However, to date the amount of TFs identified is still limited compared to the diverse volatile terpenoids. In this study, the integration analysis of DEGs and DCVOCs during floret development yielded great numbers of correlations between DEGs and DCVOCs (Fig. 4C). Although DEGs from volatile biosynthesis pathway appear not significantly correlated with VOCs, MYB TFs were found with positive correlations to the dominant volatile terpenoids production during floret development (Fig. 4C–E). Previous studies mostly found that multiple R2R3-MYB TFs are involved in regulating genes in floral volatile benzenoid/phenylpropanoid (FVBP) biosynthesis [63, 64]. Recent research report MYB TFs were also involved in regulating terpenoid emission in Hedychium coronarium, Lilium sp., and Freesia hybrida [23, 34, 65, 66]. Our results revealed four MYB genes (Chr13HA2455, Chr10HA618, Chr06HA599, and Chr03HA28) with varied associations with mono- and sesquiterpenes in C. alismatifolia during floret development (Fig. 4E). Two MYB genes, Chr13HA2455 and Chr10HA618, were observed with a broad association with both mono- and sesquiterpenes, suggesting that they could associate with the regulation of typic terpenoids. While mono- and sesquiterpenes are mainly synthesized from two independent but compartmentally separated pathways located in plastids for MEP and cytosol for MVA pathways [18, 22]. TFs are generally involved in the biosynthesis of mono- and sesquiterpenes-related volatiles by activating gene transcription in the MEP and MVA pathways. In this study, how the MYB TF genes (Chr13HA2455 and Chr10HA618) participate in the regulation of biosynthesis genes of mono- and sesquiterpenes remains to be further explored. It is noteworthy that we verified the association patterns between terpenoids and identified genes in both different floret development of “CMP” as well as eight C. alismatifolia cultivars (Fig. 5). The result found a similar association pattern between one MYB gene (Chr03HA28) and terpenoids during flower development was revealed across different cultivars (Fig. 5B and C). While the associations between identified TPS genes and terpenoids in different C. alismatifolia cultivars were not observed during flower development of “CMP.” The inconsistent findings on TPS and MYB genes in different cultivars and flower development indicate that MYB genes may have more comprehensive regulation functions than the specific TPS genes.

## Materials and method

### Plant growth and sample collection

The materials used in current study were donated from Guangdong Key Laboratory of Ornamental Plant Germplasm Innovation and Utilization, Institute of Environmental Horticulture, Guangdong Academy of Agricultural Sciences, China. Eight *C. alismatifolia* cultivars were examined, including *C*. *alismatifolia* cv. “Chiang Mai Pink” (CMP), “Yu Ki” (YK), “Snow White” (SW), “Dawn” (Da), “Siam Scarlet” (SSc), “Siam Shadow” (SSh), “Hong Yan” (HY), and *C. hybrida* “Siam Shadow” (SSha). The plants were grown in the greenhouse at Shenzhen Institute of Agricultural Genomics, Chinese Academy of Agricultural Sciences. The florets of eight cultivars were collected at the blooming stage (Fig. 1A). In addition, the florets of “CMP” were sampled at four different flowering stages (Fig. 3C): closed bud (S1), initial flowering (S2), blooming flower (S3), and withered flower (S4). All fresh samples were frozen immediately after sampling and stored at −80°C for further analysis. Three to six replications were collected for each sample, and 5–10 florets from three individual plants were mixed as a replication.

### Metabolite profiling on VOCs by gas chromatography-mass spectrophotometer (GC–MS)

All floret samples were ground into a fine powder with liquid nitrogen. 500 mg of the powder were immediately transferred into a 20 mL head-space vial (Agilent, Palo Alto, CA, USA), containing saturated NaCl solution and 10 μL of Methyl 2-Hydroxybenzoate-3,4,5,6-D4 as internal standard. The solid phase microextraction (SPME) was used to extract VOCs. Each vial was placed at 60°C for 5 min, then a DVB/CWR/PDMS fiber (Ø120 μm, Agilent, Palo Alto, CA, USA) was installed and exposed to the headspace of each sample for 15 mins to extract VOCs. Three and six replications were used for each cultivar and developmental stage, respectively.

For metabolite profiling, a GC–MS platform (8890-7000D, Agilent, Palo Alto, CA, USA) equipped with a 30 m × 0.25 mm × 0.25 μm DB-5MS (5% phenyl-polydimethylsiloxane) capillary column, was used for the identification and quantification of VOCs. After SPME, the fiber coated with VOCs was injected into the port of the GC apparatus for desorption of the VOCs at 250°C for 5 min in split-less mode. The injector temperature was kept at 250°C and the detector at 280°C. Helium was used as the carrier gas at a 1.2 mL/min linear velocity. The oven temperature was programmed from 40°C (3.5 min), increasing by 10°C/min to 100°C, by 7°C/min to 180°C, and by 25°C/min to 280°C, and held for 5 min. Mass spectra were recorded in electron impact (EI) ionization mode at 70 eV. The quadrupole mass detector, ion source, and transfer line temperatures were set at 150, 230, and 280°C, respectively. The MS with single ion monitoring (SIM) mode was set up for the identification and quantification of analytes against the NIST2011 and Metare’s metabolite database (MWDB, Metware Biological Science and Technology Co., Ltd. Wuhan, China). The relative contents of identified VOCs were calculated as the ratio of the peak area of each VOC to that of internal standard and sample weight. The identified metabolites were classified based on their chemistry structure. The odor of each metabolite was annotated according to the description in different online platforms, Perflavory Information System (http://perflavory.com/) and The LRI & Odour Database (http://www.odour.org.uk/).

### RNA extraction, sequencing, and analysis

Total RNA was extracted from powder samples of florets using a Total RNA kit (Tiangen, Beijing, China) according to the manufacturer’s protocol. RNA extracts were quality-controlled using a Nano Photometer spectrophotometer (IMPLEN, CA, USA), gel electrophoresis, and Agilent 2100 Bioanalyzer system (Agilent Technologies, CA, USA). The mRNA was purified from total RNA using poly-T beads for cDNA synthesis. After synthesis, RNA sequencing was performed using the DNBSEQ-T7 (MGI), and 150 bp paired-end reads were generated. The raw reads were quality-controlled and cleaned using fastp v0.20.1 [67]. The cleaned reads were aligned against the haplotype-resolved genome of *C. alismatifolia* [68] using STAR v2.7.10a [69], and the Fragments Per Kilobase Million (FPKM) were obtained using RSEM 1.3.3 [70]. A criterion of average FPKM >1 across eight cultivars or different developmental stages of “CMP” floret was used to identify the expressed gene in the floret.

### Terpene synthase activity assay

TPS enzyme activity assay was performed following the previous research [68]. Briefly, the coding sequence of selected *TPS* genes was inserted into the protein expression vector pCold-TF. 100 μg purified protein of the corresponding gene was incubated in a 300 μL of standard reaction mixture with 2 mM (*E*, *E*)-FPP (farnesyl pyrophosphate) as substrate at 30 for 2 hours. The volatile products were collected using a 75 μm DVB/CAR/PDMS fiber (Sigma-Aldrich) and then detected in an Agilent 5975–6890 GC–MS platform (Agilent, Technologies. Inc. CA, USA). The empty vector (pCold-TF) was used as a negative control. Data acquisition, peak detection, and metabolite annotation were performed using Xcalibur v4.1 software (Thermo Fisher Scientific, USA) against NIST2017 standard mass spectra library.

### Data analysis

The proportion of each metabolite class was calculated as the ratio of the total ion content of the VOCs with the corresponding metabolite class to the total ion content of all identified VOCs. The intensity of floral odors was calculated as the total ion content of the VOCs with the corresponding odor. The odor with the highest intensity was selected as the reference odor, and the relative intensities of other odors were calculated against the reference odor. Principal component analysis (PCA) was performed on identified metabolites and genes using *pca* function provided in “factoextra” package [71]. The differentially changed VOCs (DCVOCs) between neighboring developmental stages (S1 *vs.* S2, S2 *vs.* S3, and S3 *vs.* S4) of ‘CMP ‘florets were obtained using a *t*-test with *q* < 0.05 and log2-transformed fold change >1 (|log_2_FC| > 1). Differentially expressed genes (DEGs) were calculated using the “DESeq2” package [72] in the R platform 4.3.2 with the threshold at *q* < 0.05 and |log_2_FC| > 1. Correlation analysis was performed between metabolites in the florets of eight cultivars and between DCVOCs and DEGs of “CMP” floret using the fast correlation functions *cor* and *corPvalueStudent* in the “WGCNA” package [73] with the “Pearson” algorithm. Weight correlation network analysis (WGCNA) was applied to construct the gene expression network based on expressed genes across eight cultivars. The signed topological overlay mapping (TOM) was created based on an adjacency matrix with power at 22. The minModulesize and cutHeight for module merge were set at 150 and 0.2, respectively, to identify gene modules. The network, based on metabolite correlations and gene co-expression, was constructed using the “igraph” package and Cytoscape software v3.10.1 [74].

## Supplementary Material

Web_Material_uhae348

## Data Availability

The RNA-seq data used in this study have been deposited into China National GeneBank DataBase (CNGBdb) under project No. CNP0005815, which is publicly accessible at https://db.cngb.org/data_resources/. The datasets of metabolite and gene expression analyzed during the current study are available from the corresponding author upon reasonable request.
