# Supplementary material for: Metabolomic and transcriptomic analyses provide insight into the variation of floral scent and molecular regulation in different cultivars and flower development of Curcuma alismatifolia
Source: Hortic Res. 2024 Dec 12;12(3):uhae348. doi: 10.1093/hr/uhae348 (PMC11890029; doi:10.1093/hr/uhae348)
Supplement: Web_Material_uhae348 [file web_material_uhae348.zip › Supplemtary Figures.pptx]

## Slide 1
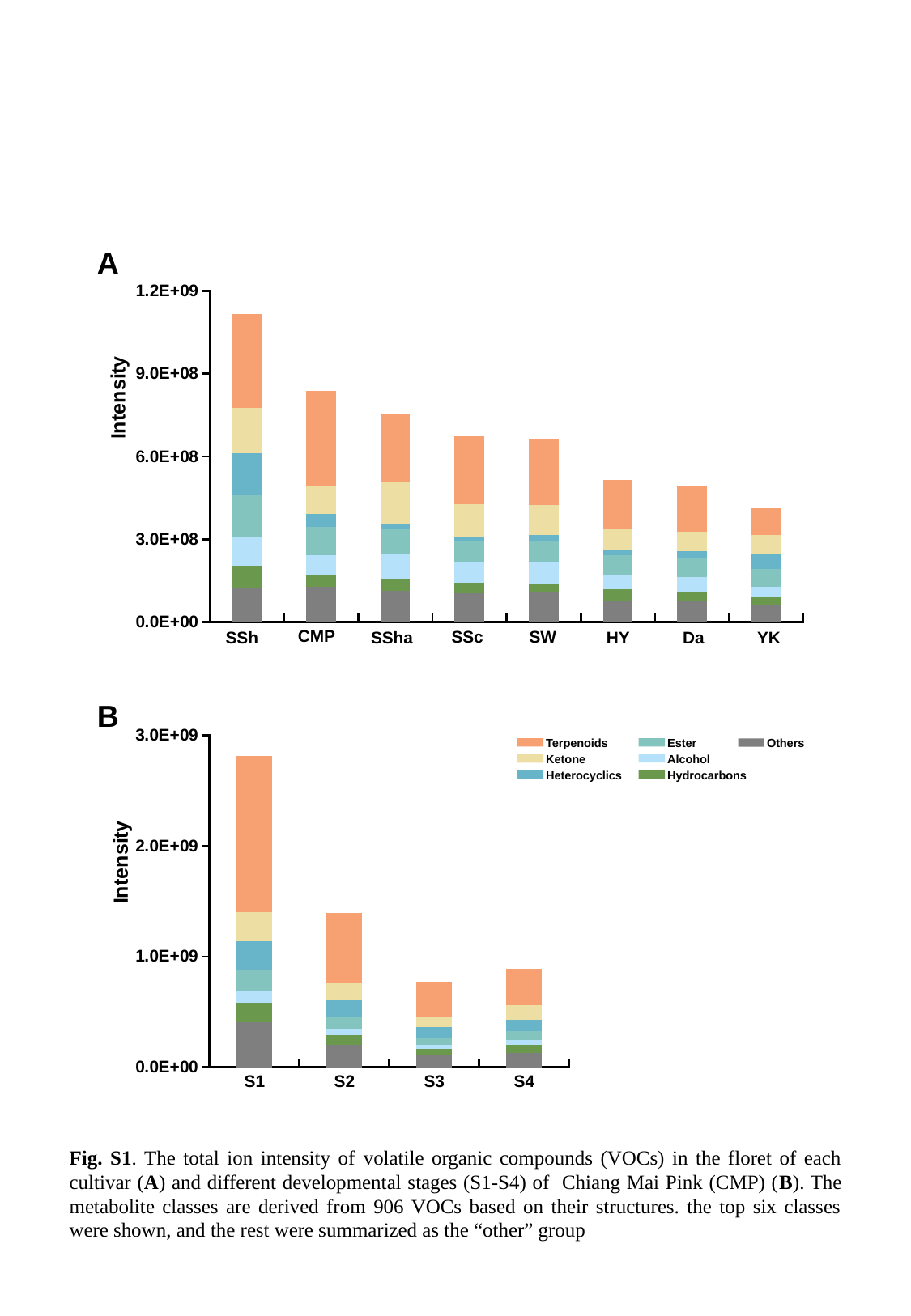

### Chart
| Category | Other | Alcohol | Ester | Heterocyclic compound | Hydrocarbons | Ketone | Terpenoids |
|---|---|---|---|---|---|---|---|
| YRY | 125840601.59920001 | 78503317.5 | 104834208.54 | 151069949.64 | 151235300.51 | 164634781.12 | 339956251.18 |
| QMF | 126841598.7098 | 41699730.28 | 74303348.397 | 100639616.03 | 46658925.724 | 103136972.92 | 343333521.61 |
| YZH | 113565962.78729999 | 44815174.094 | 90448480.389 | 89263417.474 | 15181644.492 | 152673240.72 | 247753151.82 |
| JL | 103756485.61860001 | 39855463.88 | 75513066.767 | 75582432.453 | 14195108.985 | 116643546.86 | 246233205.74 |
| BXGZ | 106202264.1491 | 32537555.22 | 80384739.111 | 75199052.76 | 20791705.145 | 108448912.91 | 236543453.5 |
| HY | 74653399.05529001 | 43540972.216 | 54726213.94 | 68756873.152 | 21651517.934 | 71727680.816 | 180947189.09 |
| LM | 76181428.2304 | 32860150.573 | 54362497.683 | 71149298.599 | 23205709.854 | 70652424.234 | 164819796.59 |
| YIKI | 60852427.89687999 | 27649947.317 | 38847239.669 | 63621281.515 | 52771856.437 | 72842103.874 | 94093728.283 |CMP
SW
SSc
SSh
SSha
HY
Da
YK
A
B
### Chart
| Category | Other | Hydrocarbons | Alcohol | Ester | Heterocyclics | Ketone | Terpenoids |
|---|---|---|---|---|---|---|---|
| S1 | 405934060.62590003 | 179651957.37 | 100030165.96 | 186772917.58 | 263592905.91 | 268542969.69 | 1411171386.1 |
| S2 | 199773142.57709998 | 90851263.363 | 58171606.223 | 107813377.94 | 145621132.39 | 159709511.07 | 633091766.9 |
| S3 | 117010367.48410001 | 45040169.5 | 39530097.76 | 68471459.514 | 94595420.145 | 93373424.016 | 313218696.36 |
| S4 | 125469988.75469999 | 77641384.274 | 43127481.423 | 81096653.349 | 101230159.24 | 128332191.25 | 335527460.91 |Ester
Terpenoids
Others
Alcohol
Ketone
Hydrocarbons
Heterocyclics
Fig. S1. The total ion intensity of volatile organic compounds (VOCs) in the floret of each cultivar (A) and different developmental stages (S1-S4) of Chiang Mai Pink (CMP) (B). The metabolite classes are derived from 906 VOCs based on their structures. the top six classes were shown, and the rest were summarized as the “other” group

## Slide 2
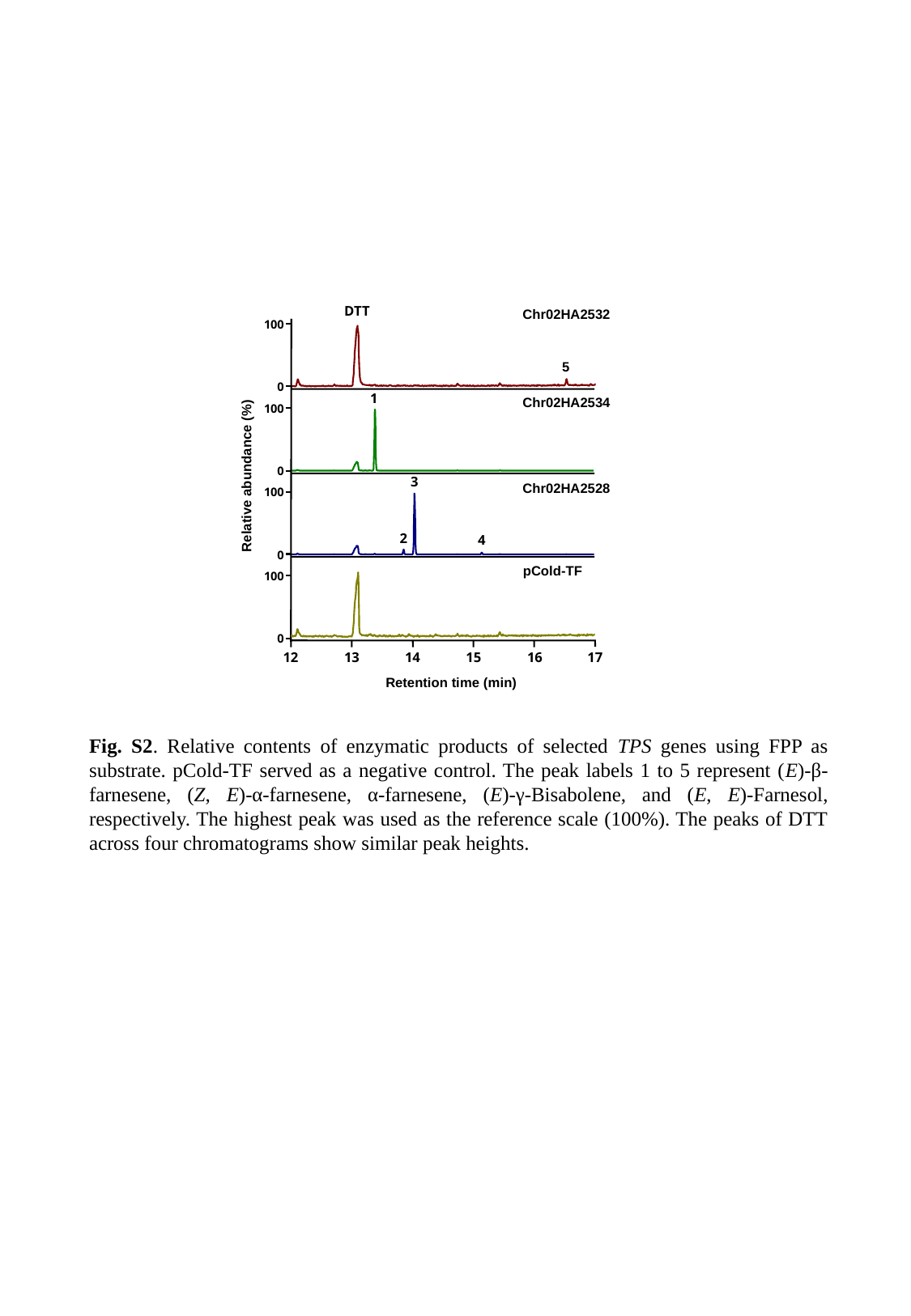

DTT
Chr02HA2532
100
5
0
1
Chr02HA2534
100
0
Relative abundance (%)
3
Chr02HA2528
100
2
4
0
pCold-TF
100
0
12
13
14
15
16
17
Retention time (min)
Fig. S2. Relative contents of enzymatic products of selected TPS genes using FPP as substrate. pCold-TF served as a negative control. The peak labels 1 to 5 represent (E)-β-farnesene, (Z, E)-α-farnesene, α-farnesene, (E)-γ-Bisabolene, and (E, E)-Farnesol, respectively. The highest peak was used as the reference scale (100%). The peaks of DTT across four chromatograms show similar peak heights.

## Slide 3
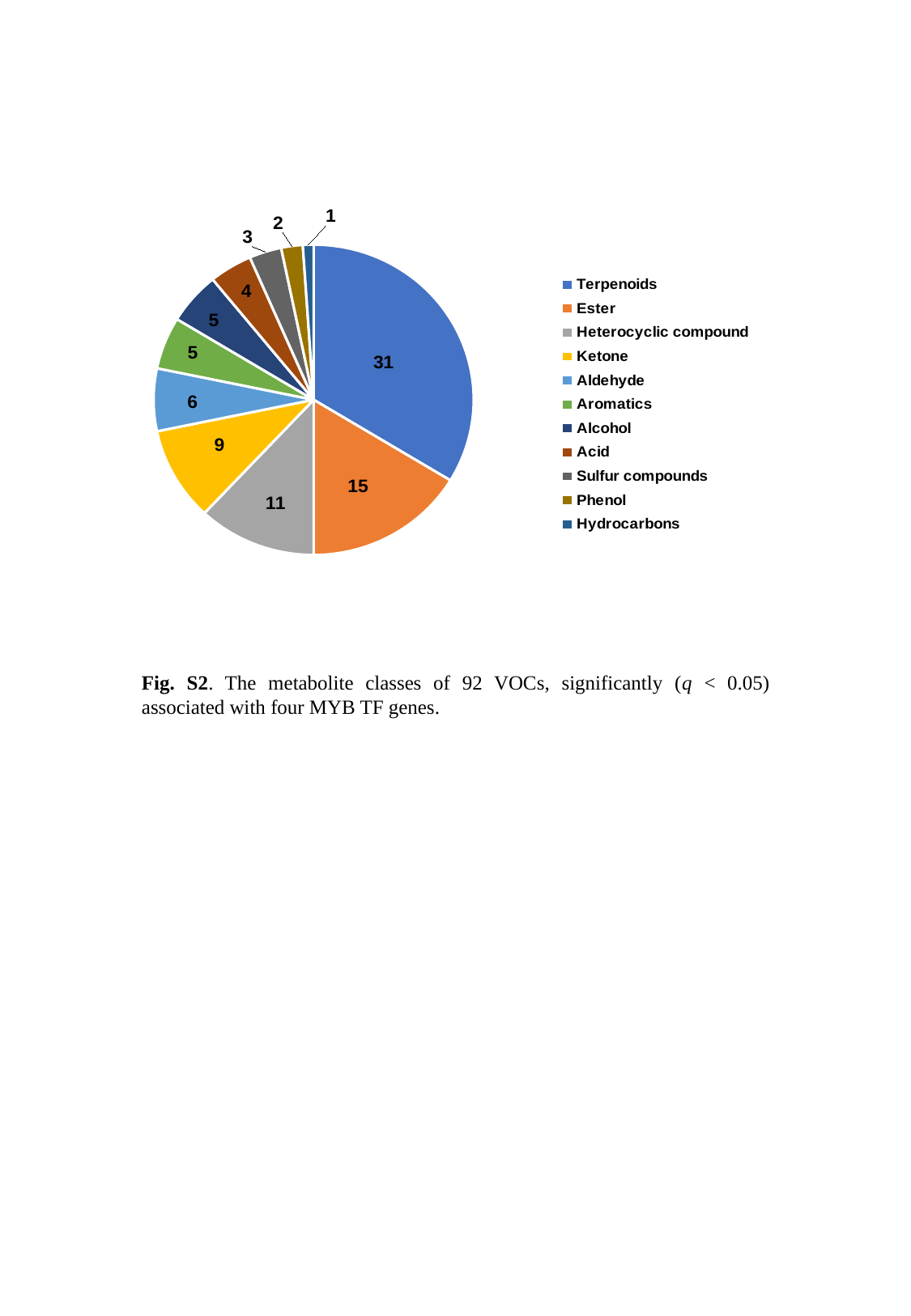

### Chart
| Category | count |
|---|---|
| Terpenoids | 31.0 |
| Ester | 15.0 |
| Heterocyclic compound | 11.0 |
| Ketone | 9.0 |
| Aldehyde | 6.0 |
| Aromatics | 5.0 |
| Alcohol | 5.0 |
| Acid | 4.0 |
| Sulfur compounds | 3.0 |
| Phenol | 2.0 |
| Hydrocarbons | 1.0 |Fig. S2. The metabolite classes of 92 VOCs, significantly (q < 0.05) associated with four MYB TF genes.
